# Ostrich oil as a fat substitute in milk‐based infant formula

**DOI:** 10.1002/fsn3.3220

**Published:** 2023-01-09

**Authors:** Mohsen Dalvi‐Isfahan, Zohreh Moammernezhad, Javad Tavakoli

**Affiliations:** ^1^ Department of Food Science and Technology, Faculty of Agriculture Jahrom University Jahrom Iran

**Keywords:** cholesterol, fatty acid composition, infant formula, ostrich oil

## Abstract

In this study, the possibility of replacing vegetable fats with ostrich oil in infant formula (IF) production was investigated. The fatty acid profile, the positional distribution of fatty acids in the triacylglycerols, the cholesterol content, and the physicochemical properties of ostrich oil were determined and compared with breast milk fat and vegetable oils. In the next step, two infant formulas were produced using ostrich oil and vegetable oils and the physicochemical properties, rheological properties, color parameters, and sensory analysis of the resultant powders were compared. The results showed that the predominant fatty acids in ostrich oil are palmitic acid, oleic acid, and linoleic acid which is similar to breast milk fat and vegetable oils. The presence of appropriate cholesterol content in ostrich oil makes it more similar to breast milk fat compared to vegetable fats. Palmitic acid was located at sn‐2 position in 15% triacylglycerol from ostrich fat, which was equal to the amount reported for vegetable fats. The incorporation of ostrich oil in infant formula production showed that there is no statistically significant difference between quality attributes of powder formulated with ostrich oil or vegetable oils. Therefore, ostrich oil can be introduced as a new source of edible oil, and addition of ostrich oil is an effective way to reduce the gap between the composition of breast milk and infant formula.

## Graphical abstract

This manuscript describes about the ostrich oil as a new source of edible oil and incorporation of ostrich oil in infant formulas to reduce the gap between the composition of breast milk and infant formula.

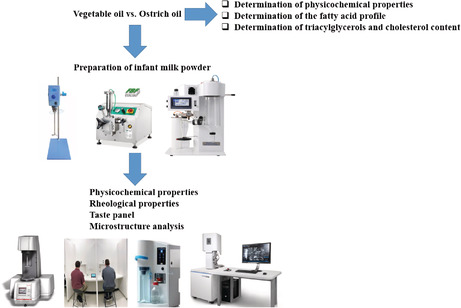

## 1 INTRODUCTION

Currently, nearly 1.5 million Iranian infants under 2 years of age are fed with infant formula (IF), so all infants consume more than 35 million cans (each 400 g) of IF per year.

Based on statistics from the Ministry of Health of Iran, 97% of children have no problem in drinking breast milk, but despite recurrent explanations and announcements that breast milk is superior to IF, and the national prohibition of advertisements for infant formulas in Iran, the statistics show that about half of Iranian infants (under the age of 2 years) consume IF (Ahmadi et al., 2019). According to the Iranian Union of Infant Formula Producers, although the processing and production of IF are operated in Iran itself, about 85% of the raw materials needed for production are supplied from European countries. Meanwhile, the largest share of the import is fat, which makes up about 30% of the weight of the final product.

Fat is one of the most important components of IF and 50% of an infant's energy needs are normally provided by this component. Nowadays, there are three important sources of fat in IF, that is, vegetable fats, bovine milk fat, and goat's milk fat. However, each fat source has its own advantages and limitations (Berger et al., 2000; Sun et al., 2016).

The main differences between breast milk fat and fat from other sources are mainly due to a number of factors, including the cholesterol content, polar lipids, the positional distribution of fatty acids in triacylglycerols, and fatty acid composition (Hokkanen et al., 2022).

The most common vegetable fats are coconut oil, corn oil, soybean oil, palm oil, sunflower oil, high oleic acid safflower oil, and low erucic acid rapeseed oil (Mendonça et al., 2017). Among the many types of vegetable oil, palm oil has the largest share of palmitic acid which is required to attain a similar level in breast milk (Hageman et al., 2019). However, the utilization of vegetable oils, especially palm oil, can be associated with some problems. For instance, human milk triglycerides contain palmitic acid primarily in the sn‐2 position, whereas infant formula fat blends contain palmitic acid primarily in the sn‐l and sn‐3 positions, which cause problems in calcium absorption and excretion by infants (Hageman et al., 2019; Nelson et al., 1998).

In addition, there have been some concerns about the existence of high levels of pollutants caused by processing palm oil, which can have negative effects on the health of newborns (IARC, 2013). Another problem with the use of vegetable oils in IF is the absence of cholesterol and the presence of plant sterols (phytosterols). In this regard, Cruz et al. (1994) reported that cholesterol synthesis in formula‐fed infants was three times higher than in breast‐fed infants. In addition, there might be a connection between cholesterol exposure early in life and the development of enzymes for cholesterol degradation and amounts of endogenous cholesterol synthesized, which results in lower cholesterol levels in individuals who were breastfed as infants (Coad et al., 2019).

Utilization of bovine milk fat and goat milk fat in infant formulas is also associated with some problems. For example, bovine milk contains higher levels of saturated fatty acids compared to human milk fat and lower levels of long‐chain unsaturated fatty acids such as oleic and linoleic acids. Meanwhile, bovine and goat milk fats contain relatively high concentrations of short‐chain length fatty acids compared with breast milk (Ceballos et al., 2009). Although medium‐chain fatty acids are very rapidly absorbed in newborns, and they do not need carnitine to be metabolized in the cell, the human milk contains <2%, and any excess could induce acidosis by the production of carboxylic acid. Another unfavorable feature of bovine and goat milk fats is the high percentage of lauric and myristic fatty acids in the oil. The possible adverse effects of C12:0 and C14:0 on cholesterol and serum lipoprotein concentrations of infants were reported by Koletzko et al. (2005). Despite suggestions and attempts to modify the structure of vegetable oil through interesterification or blending vegetable oils and bovine milk fat, none of the said methods have provided a suitable oil that can imitate all the characteristics of human breast milk and have a reasonable price at the same time (Hageman et al., 2019; Miles & Calder, 2017).

In recent years, there has been an increase in the breeding of ostriches in Iran, which has made the country become the second leading producer of ostriches in the world, after South Africa. One of the important wastes during animal slaughtering is carcass fat, which includes about 35% of the carcass weight. This oil is rich in unsaturated fatty acids and contains omega‐3, omega‐6, omega‐7, and omega‐9. The seven predominant fatty acids of ostrich oil are oleic, palmitic, palmitoleic, linoleic, stearic, alpha‐linolenic, and gamma‐linolenic acids (Basuny et al., 2017; Dalvi & Daraei, 2012; Gavanji et al., 2013; Horbańczuk et al., 2004).

The purpose of the current study is undertaken to investigate the use of ostrich oil as a new type of fat for infant formula for the first time. Accordingly, in the first step, physicochemical characteristics and structure of fatty acid and triglycerides of the oil are determined and compared with the breast milk. In the second step, two infant formulas were produced using ostrich oil and vegetable fats, and comparisons will be made regarding their chemical, physical, rheological, and sensory properties. The results of this research can suggest a new, cheap and suitable source of fat for the production of infant formulas, especially in developing countries.

## 2 MATERIALS AND METHODS

Ostrich oil in this study was obtained from Makian Raika Oil Company (Isfahan, Iran). It was extracted by the wet rendering method, followed by bleaching and deodorization processes. Meanwhile, blended vegetable oils formulated for infant formula by the AAK company (Netherlands) were provided by Pegah infant formula Co. Iran. Vegetable oil is a fat blend for baby milk that consists of various ingredients such as palm, rapeseed, sunflower oil, lecithin (E322), and ascorbyl palmitate (E304) and complies with the Directive 2006/141/EC on infant formulae and follow‐on formulae. It is important to note that, regardless of the main fat source, arachidonic acid (ARA) and docosahexaenoic acid (DHA), are always added separately to the product as a supplement.

In this research, however, none of these two fatty acids were added to the infant formulas. To produce IF, the skim milk powder and other raw materials were obtained from Pegah Infant Formula Co. (Shahrekord, Iran). These ingredients were demineralized whey (Lactalis), lactose (MEGGLE), and a premix of minerals and vitamins (Vitablend Nederland BV).

### 2.1 Physicochemical properties of fat

The physical and chemical properties of both oils (ostrich and vegetable oils) were determined in three replicates, and the following items were measured. Iodine value, acid value, and saponification number were measured according to AOAC 920.158 (Hanus method), Cd 3d‐63 and 920,160 methods, respectively (AOAC, 2005). Meanwhile, the amount of peroxide was determined according to the method previously reported by Tavakoli et al. (2019).

### 2.2 Determination of the fatty acid profile of the oil

The fatty acid composition of the oil sample was determined by gas chromatography according to the method by Eder (1995). Briefly, oil solutions in hexane (0.3 g in 7 ml) were diluted with 2 ml of methanolic potassium hydroxide at 50°C for 10 min. Fatty acid methyl esters (FAME) were detected using chromatography (Hewlett‐Packard, CA, USA) HP‐5890 equipped with silica glass CP‐FIL88 capillary column, with 60 m length in 0.22 mm I.D., 0.2 μm film thickness, and flame ionization detector (FID). Nitrogen gas was used as a carrier gas with a flow rate of 0.75 ml/min. The oven was maintained at a temperature of 198°C. The injector and detector were set at a temperature of 250°C.

### 2.3 Determination of triacylglycerols and cholesterol

The sample was prepared according to the method specified in ISO (ISO/TS 17383). SHIMADZU gas chromatography device (Nexis 2030, Japan) was equipped with a flame ionization detector (FID), RESTEK Rtx‐65 capillary column, 60 m long, 0.25 mm in diameter, along with an injector (PTV). In this device, the temperatures of the injector and detector were 340 and 370°C. Helium was used as a carrier gas with a purity of 99.99%, and with a flow rate of 4 ml/min.

Cholesterol measurement was performed by gas chromatography SHIMADZU (Nexis 2030, Japan). Briefly, the oil was saponified using 2.5 M potassium hydroxide solution at 60°C. Then, the unsaponified part of the sample was separated on the top of the container with n‐hexane. In the next step, the samples were derivatized with 50 μl of trifluoroacetamide with 1% of trimethylchlorosilane at 60°C. The temperatures of the injector and the detector were adjusted to 320 and 255°C, respectively, and hydrogen gas was used (99.99% purity) at a flow rate of 1.5 mm/min (Izadi et al., 2015).

### 2.4 Preparation of infant milk powder

Percentages of ingredients used in the infant formula are shown in Table 1. All powder materials were mixed together firstly, and then distilled water (45°C) was added to all powder materials. Stirring was continued using a slow stirrer until all powder materials were dissolved completely. Then, oil and emulsifier (lecithin) were gradually added to the ingredients. Mixtures were pasteurized at 80°C for 20 s in a laboratory heat exchanger immediately after dispersion. In order to homogenize the samples, the laboratory homogenizer (Homolab 2.50; FBF ITALIA) was used. The homogenization operation was done in two stages with primary and secondary pressures of 300 and 80 bar, respectively. The homogenized samples were further converted into powder by a laboratory spray drier, Dorsa Behsaz Co., with inlet and outlet temperatures of 145 and 75°C, respectively. After the powders were collected and reached to ambient temperature, they were stored in dark‐colored glass containers for further analysis.

**TABLE 1:** Percentages of ingredients used in the infant formulation

Ingredients	Percentage (%)
Demineralized whey	43.67
Oil	27.25
Lactose	12.28
Skim milk powder	16.5
Mineral premix	0.2
Vitamin premix	0.1

### 2.5 Chemical analysis of milk powder

The dry matter was determined by drying of 5‐g sample in a convection oven (Memmert) for 10 h at 90°C. The moisture content was calculated as the weight loss after drying. A nitrogen‐to‐protein conversion factor of 6.25 was used to calculate the protein content of a milk powder from the nitrogen content determined by Kjeldahl method.

Fat content was measured using the Gerber method, and total ash content was calculated by burning the samples at 600°C in an electric furnace. Total lactose content was calculated by weight difference (Kelly et al., 2016). The peroxide value of milk powders was determined 30 days after production according to the method reported by Tavakoli et al. (2019).

### 2.6 Rheological properties of infant milk powder

Rheological properties were determined using an MCR 302 rheometer from Anton Paar (Austria), which had a temperature control system. Three milliliters of milk powder solution was added to 30 g of distilled water and stirred. After the thorough mixing, the solution was poured into the chamber of the rheometer cylinder for 2 min. In addition, in order to investigate the effects of processing condition such as evaporation stage, IF formula with 36 °Brix was prepared and introduced to the rheometer. Then, the characteristics of flow behavior, apparent viscosity, and shear rate 0–1000 s^−1^ were measured at 25°C (Alatalo & Hassanipour, 2020). The power‐law model was used to describe the rheological properties of the solutions.

$$\tau =m\left(\gamma^{n}\right)$$

where τ is the shear stress (Pa), γ is the shear rate (s^−1^), while the parameters m and n are the consistency index and the flow behavior index, respectively.

### 2.7 Microstructure of milk powder with SEM

The morphology of the particles was evaluated with a VEGA‐3 scanning electron microscope manufactured by Tescan, with a voltage of 5 kV and a magnification of 5, 10, and 20 μm (de Oliveira et al., 2021). SEM images were analyzed in triplicate using Image J v1.50a software (National Institutes of Health) to determine particle size.

### 2.8 Determining the color of powdered milk with color indexes L* a* b*

Color analysis was obtained by taking pictures using a digital camera of milk powder poured onto the plate and pressed to create a smooth surface. Then, the plates were placed in a wooden box that was equipped with two lamps which reproduced the qualities of natural daylight. The images were taken with a Canon PowerShot A495 color digital camera with a resolution of 10 megapixels at different magnifications. Photoshop® CS 7.0 software was used for determining L*, a*, and b* parameters (Afshari‐Jouybari & Farahnaky, 2011).

### 2.9 Sensory evaluation

The sensory evaluation of the samples was evaluated by the preference test (hedonic scale) (Guinard, 2000). On the hedonic scale, liquid powdered milk was stored in the refrigerator for 24 h. Fifteen tasters from students of food science and technology at Jahrom university were selected for sensory evaluation after providing them with preliminary training. Thirty milliliters of the drink (20°C) was given to each taster. Sensory tests were conducted according to the 5‐point hedonic scale (1‐dislike extremely; 2‐dislike slightly; 3‐neither like nor dislike; 4‐like slightly; 5‐like). The following attributes of flavor, color, and overall acceptance of the drink were evaluated by the tasters (Buono et al., 1990).

### 2.10 Statistical analysis

The experiment was carried out in three replicates. Tables and figures presented data that were reported as mean ± standard deviation. Data were analyzed by SPSS software package (version 17.0; SPSS). The data were evaluated using Student's *t*‐test to compare two groups (ostrich vs. vegetable oils).

## 3 RESULTS AND DISCUSSION

### 3.1 Physicochemical properties of oil

Table 2 shows the physicochemical properties of ostrich oil and vegetable oils. As can be seen, acid value and peroxide value were in the normal range, which indicates suitable processing and storage conditions for both oils. Other physicochemical indicators of the two oils were also within the standard range. For example, according to the AAK company standard, the limit of iodine number for infant fat should be in the range of 70–90. Here, an interesting point is the low melting point of ostrich oil compared to the melting point of many other animal fats. For instance, the melting point of bovine fat and sheep fat is 40–48 and 44–52°C, respectively, which are much higher than that of ostrich oil. The reason for this difference is the higher content of saturated fatty acids, with a higher proportion of stearic acid and a lower proportion of palmitoleic and oleic acids in other animal fats compared to ostrich oil (Daley et al., 2010).

**TABLE 2:** Chemical characteristics of ostrich oil and vegetable oil sample

Parameter	Ostrich oil	Vegetable oils
Acid value	0.02 + 0.01	0.035 + 0.01
Peroxide value (meq O_2_/Kg)	0.35 + 0.05	0.25 + 0.04
Iodine value	76 + 4.1	79 + 5.3
Saponification value	200 + 10	190 + 8.5
Melting point (°C)	32 + 1.5	29 + 1.6
Refractive index	1.42 + 0.2	1.39 + 0.35

### 3.2 Fatty acid profile

The predominant fatty acids in ostrich oil and breast milk fat were similar to each other and include oleic acid, palmitic acid, stearic acid, and linoleic acid (Table 3). A higher level of palmitic acid was found in ostrich fat compared to human milk fat (31.73% vs. 21.93%). The amount of oleic acid in ostrich oil and breast milk fat was nearly similar (38.52% vs. 36.3%).

**TABLE 3:** Fatty acid composition (g 100 g^−1^ fatty acids) of human milk, vegetable oil, and ostrich oil

Fatty acids		Human milk (Hageman et al., 2019)	Ostrich oil	Vegetable oil
Short‐chain fatty acids				
C4:0	Butyric	ND	ND	ND
Medium‐chain fatty acids				
C6:0	Caproic	0.39	ND	ND
C8:0	Caprylic	0.19 (0.09–0.24)	ND	ND
C10:0	Capric	1.29 (0.83–1.63)	ND	ND
Long‐chain fatty acids				
C12:0	Lauric acid	5.98 (4.15–8.33)	0.22	0.13
C14:0	Myristic acid	6.44 (4.98–9.38)	0.9	0.637
C14:1	Myristoleic acid	0.18	ND	ND
C15:0	Pentadecanoic acid	0.25 (0.16–0.32)	ND	ND
C16:0	Palmitic acid	21.93 (15.43–25.62)	31.73	29.28
C16:1	n‐7 Palmitoleic acid	1.98 (1.65–2.31)	7.3	0.168
C17:0	Heptadecanoic acid	0.29 (0.22–0.33)	0.08	ND
C18:0	Stearic acid	7.37 (5.58–9.52)	6.74	4.07
C18:1 n‐9	Oleic acid	36.30 (28.93–41.69)	38.52	41.42
C18:2 n‐6	Linoleic acid	13.99 (10.16–16.59)	13.02	17.97
C18:3 n‐3	Alpha‐linolenic acid	0.76 (0.49–1.05)	0.95	1.89
C20:0	Arachidic acid	0.21 (0.14–0.31)	ND	ND
C20:1 n‐7	Paullinic acid	ND	ND	1.94
C20:3 n‐6	Dihomo‐gamma‐linolenic acid	0.38 (0.29–0.52)	0.27	0.51
C20:5 n‐3	Eicosapentaenoic acid	0.09 (0.05–0.13)	ND	0.17
C22:0	Behenic acid	0.09 (0.05–0.13)	0.23	0.25
C20:4 n‐6	Arachidonic	0.47 (0.37–0.64)	ND	ND
C24:0	Tetracosanoic acid	0.07 (0.03–0.16)	ND	0.12
C22:6 n‐3	Docosahexaenoic acid	0.28 (0.18–0.42)	ND	0.15
C24:1 n‐9	Nervonic acid	ND	ND	0.24
Total saturated fatty acids		44.48	40.04	34.51
Total monounsaturated fatty acids		38.45	45.47	44.57
Total polyunsaturated fatty acids		15.97	13.97	20.85
Total unsaturated fatty acids		54.42	59.44	65.43

Monounsaturated fatty acids (MFA) alone comprise almost a third of the lipids in human breast milk and play an important role in the structure of nerve fibers (myelin) and also supplying the energy required by an infant. The linoleic acid content in both oils was almost equal to 13% (Barreiro et al., 2018; Cruz‐Hernandez et al., 2013). In the vegetable oils, the amount of these three fatty acids (palmitic, oleic, and linoleic) was 29%, 42%, and 18%, respectively, which means that all three were higher than the level of fatty acids in breast milk.

Stearic acid was also found at similar levels in breast milk and ostrich oil (6%–7%), while it was about 4% in vegetable oil. The alpha‐linolenic acid content in both ostrich oil and breast milk was also equal and included about 1% of the total fatty acids. It is worth noting that variations in the fatty acid profile of human breast milk can be very wide and depend on several environmental and maternal genetic factors (Pham et al., 2020).

In ostrich fat, palmitoleic acid (C16:1 n‐7) content comprises nearly 7.3% of the oil, which is greater than the amounts observed in breast milk (2%) and vegetable oils (0.2%) (Morgan & Dhayal, 2010). There have been indications that palmitoleic acid has beneficial effects on insulin sensitivity, cholesterol metabolism, and homeostasis. Also, there were positive reports on the effectiveness of this fatty acid in reducing LDL and increasing HDL (Mozaffarian et al., 2010). However, no research has been conducted on the positive or negative effects of this fatty acid on infant health.

The main difference between the fatty acid profiles of breast milk and ostrich oil was the higher level of lauric and myristic acids in breast milk. The contents of these fatty acids in human breast milk are 5% and 6%, respectively, while in ostrich oil they make up 0.2% and 1% of all fatty acids, respectively. In the vegetable oil sample, the amounts of these two fatty acids were 0.13 and 0.63%, respectively, which were much lower than the amounts in ostrich fat and breast milk fat. Nonetheless, the low‐level contents of these two fatty acids in ostrich oil can have positive effects because as Koletzko et al. (2005) reported, these two fatty acids can increase the concentrations of cholesterol and lipoprotein in the newborn's serum. However, further research needs to be conducted to assess the impact of lauric and myristic in infant nutrition.

The total amount of saturated fatty acids, monounsaturated fatty acids, and polyunsaturated fatty acids were, respectively, 40.04, 45.45, and 13.97 in ostrich oil. However, in human breast milk, approximately 44.48% of fatty acids are saturated, 38.45% are cis‐monounsaturated, and 15.97% are polyunsaturated fatty acids. Although these values can be markedly influenced by maternal diet and, to a lesser degree, by stage of lactation, in vegetable oils, they were 34.51, 44.57, and 20.85, respectively. The ESPGAN committee also suggested a ratio of linoleic acid to alpha‐linolenic acid between 5 and 15, by analogy with what is observed in human milk. Since this ratio for both ostrich oil and vegetable oil was obtained as 13.7 and 9.5, respectively, it shows both oils are within the standard range. As explained before, regardless of the main source of fat used, ARA and DHA are always added separately to the selected formula as a supplement, so it can be concluded that ostrich oil could be potentially added together with DHA and ARA for producing infant formula. Since the fatty acid profile of ostrich oil substantially depends on various genetic and environmental factors (e.g., ostrich diet and age), it is necessary to standardize the oil before incorporating it into infant formulas.

### 3.3 Triacylglycerol structure in ostrich oil and cholesterol content

Most of the triacylglycerides in human milk have palmitic acid (16:0) or oleic acid (18:0) at position 2 of the molecule (sn‐2). The unusual position of palmitic acid in breast milk triglycerides can play an important role in the health of the newborn from various aspects, e.g., normal absorption of fat and calcium, bone health, normal gut microbiota, and improved stool consistency (Havlicekova et al., 2016; Pham et al., 2020). The distribution of palmitic acid in the sn‐2 position in human breast milk, cow's milk, and vegetable fats has been reported in the available literature (Bracco, 1994; Hageman et al., 2019). Their results showed that, on average, 79%, 42.5%, and 15% of the total palmitic acid in breast milk, cow's milk, and vegetable oil were in the sn‐2 of glycerol. The result of present study indicated that about 15% of palmitic acid in ostrich oil is located in sn‐2, which is equal to blended vegetable oil formulated for infant formulas (Table 4).

**TABLE 4:** Positional distributions of fatty acids in triacylglycerols of ostrich oil

No.	TAG	Percentage (%)
1	PPP	3.541588
2	PPS	1.041644
3	PPO	10.48222
4	MOO	2.192934
5	PPoO	6.15118
6	PLP	6.348544
7	PLPo	4.265256
8	MLO	1.17322
9	PSO	4.013069
10	POO	16.77594
11	PLS	3.048178
12	PoOO	9.210322
13	PLO	7.861668
14	PoLO	5.065677
15	PLL	1.513124
16	SSO	0.427622
17	SOO	3.037213
18	OOO	5.789345
19	SLO	3.256507
20	OOL	0.635951
21	OLL	3.168789
C14:0	M	
C16:0	P	
C18:1	O	
C18:2	L	
C18:0	S	
C16:1	Po	

### 3.4 Cholesterol content

The cholesterol content in the ostrich oil sample was about 29 mg per 100 g of oil, which is lower than other birds (chicken). In addition, the level of cholesterol content in the present study was lower than those reported previously by Horbańczuk et al. (2004). Differences existing between the result of this work and Horbańczuk et al. (2004) study could be due to the diet, age of the animal, and especially the applied process conditions. As mentioned in the materials and methods section, the ostrich oil used in this research was produced by the wet rendering method and was then subjected to deodorization processes. Removal of sterols in the oil deodorization stage has been reported by Maniet et al. (2019). The degree of removal depends on the physical properties of the components: vapor pressure, temperature, and volume of steam passing through the oil. The amount of cholesterol in human breast milk progressively changes with the stage of lactation. The concentration of cholesterol is highest in the colostrum and gradually decreases with the progress of lactation. Cholesterol concentrations in adult human breast milk have reportedly ranged between 101 and 233 mg/L (Yang et al., 2022). As the present results show, the cholesterol content in refined ostrich oil was slightly higher than that of breast milk.

### 3.5 Chemical properties of powdered milk

The next stage of this study was the production and comparison of milk powders produced from the two different sources of oil (ostrich oil vs. vegetable oils). Table 5 shows the chemical composition of the powder produced from these two formulas. As can be seen, the chemical composition of the two corresponding powders confirms the aptitude of the production method (Jiang, 2014). The peroxide values for both samples were also in the acceptable range after 30 days of storage at room conditions (Table 5).

**TABLE 5:** Chemical composition of milk powder produced by ostrich oil and vegetable oil

Analysis (%, wet basis)	Milk powder (ostrich)	Milk powder (vegetable oil)
Protein	12.25 ± 1.2	13.78 ± 1.2
Fat	28.15 ± 1.7	28.26 ± 1.1
Moisture	2.15 ± 0.25	1.95 ± 0.35
Ash	1.77 ± 0.35	1.9 ± 0.42
Carbohydrate^a^	55.73	53.88
Peroxide value 30 days after production (meq O_2_/Kg)	0.36 ± 0.12	0.27 ± 0.21

*Note*: Values represent means of three determinations ± standard deviations.

^a^ Carbohydrate percentage was calculated as 100−(Moisture + fat + protein + ash).

**FIGURE 1:**
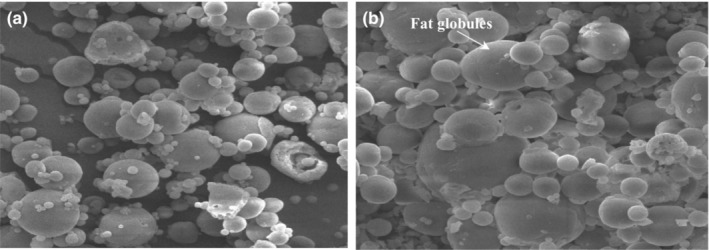
Scanning electron microscopy of infant formula (10 μm) (a) ostrich oil and (b) vegetable oils.

In addition, in order to compare the morphology of powdered infant formulas, SEM analysis was performed (Figure 1) and the particle size was determined using imageJ software.

The results showed that the average size of fat globule in the milk powders produced by two different oil sources (ostrich oil vs. vegetable fats) was 1.14 ± 0.24 and 1.15 ± 0.17 μm, respectively. Michalski et al. (2005) reported that the diameter of fat globules in IF is much smaller than the diameter of fat globules in human breast milk (4 microns vs. 0.4 μm). In fact, this is an important factor in the digestion and absorption of milk fat. The size of fat particles in infant formula depends substantially on the pressure and number of homogenization steps.

Table 6 shows the fat‐soluble vitamin content in the IF.

**TABLE 6:** Fat‐soluble vitamin content in the IF

Composition	Unit	Per 100 g	Per 100 ml
Vitamin A	IU	1831	234.4
Vitamin D	μg	8.5	1.1
Vitamin E	mg‐αTE	8	1
Vitamin K	μg	35	4.5

### 3.6 Evaluating the effects of shear rate on the rheological behavior

When replacing raw material, it is necessary to investigate its effect on the rheological changes of the product. Variations in shear rate versus shear stress were studied in two different ranges of shear rate according to the preparation and production stages.

**FIGURE 2:**
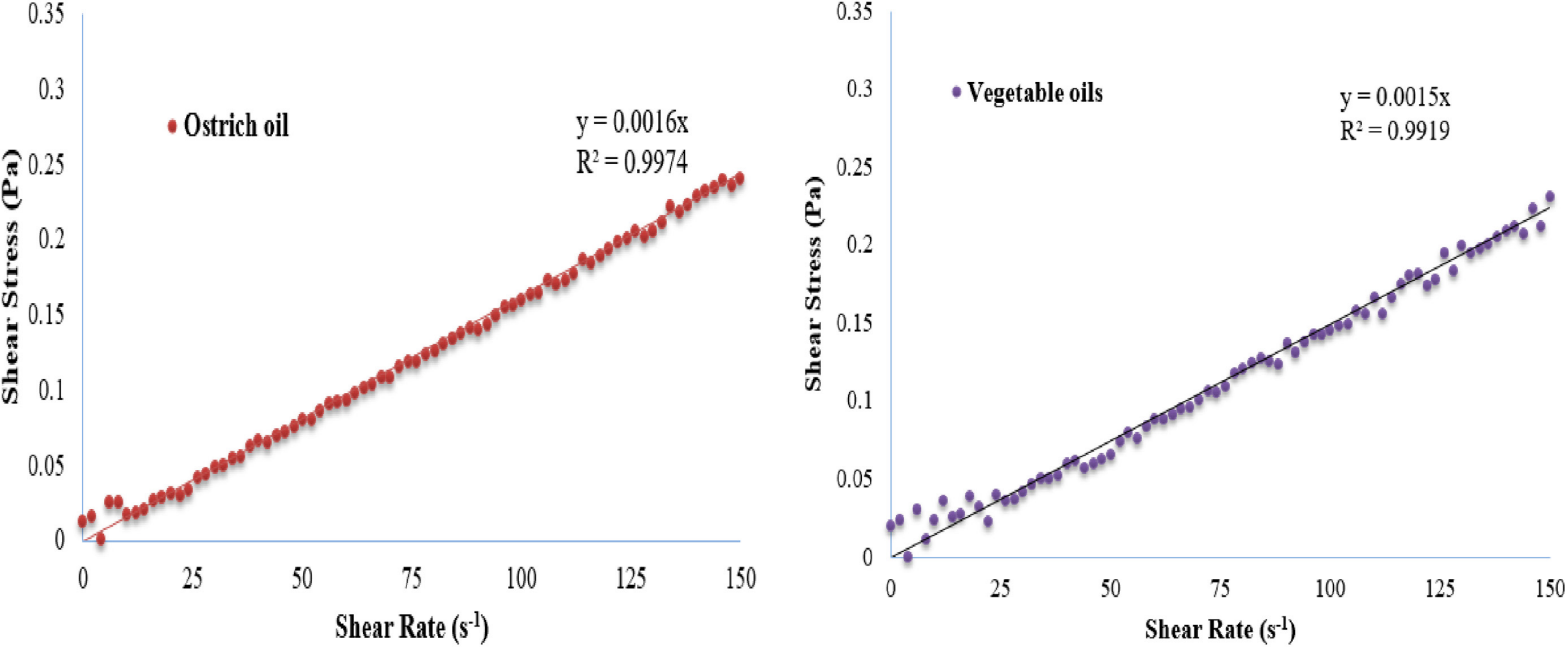
Changes in shear stress as a function of shear rate in the range of 0–150 Hz.

In the preparation of milk powders, the watery formula may cause aspiration during swallowing or cause reflux of esophageal contents into the stomach. In contrast, too concentrated infant formulas may interfere with the infant's breathing. Therefore, it would be crucial to understand the rheological properties of IF for proper swallowing and, also, for designing formula suitable for infants who struggle with dysphagia (Almeida et al., 2011; Yoon & Yoo, 2017). Both milk samples were prepared according to the label directions for use. Figure 2 shows the changes in shear stress as a function of shear rate in the range of 0–150 Hz. As can be seen, both samples exhibited Newtonian behavior (*R* ^2^ > 99%). There were insignificant differences between the viscosity of the two milk samples which were made with either ostrich oil or vegetable oil sources.

Further to the importance of rheological properties of the product and its effect on the final consumer, the effect of this change should also be considered in the production and manufacturing processes. During the production of infant formula by the wet process, two important operations of homogenization and spray drying occur, which have high shear rates (Vignolles et al., 2009). To evaluate the effect of oil replacement on the processing stages, milk samples (Bx = 36) were prepared and introduced to the rheometer.

**FIGURE 3:**
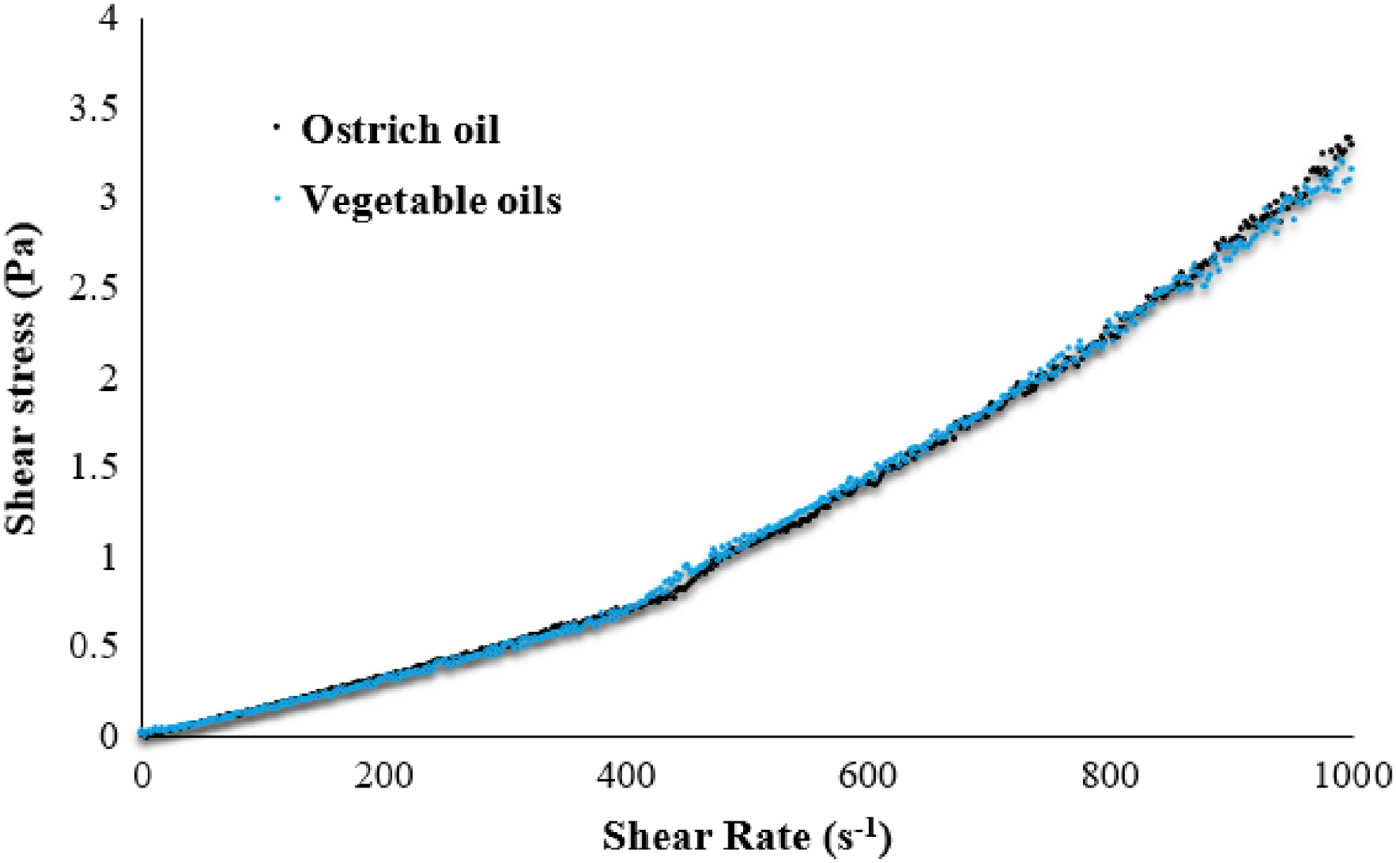
Changes in shear stress as a function of shear rate in the range of 0–1000 Hz.

Figure 3 shows the effect of a high shear rate (0–1000) on shear stress. The rheological behavior of both solutions was approximated by a power‐law model. The result showed that the flow behavior index was 5.1 and 4.8 for ostrich oil and vegetable oils, respectively. Since both values were greater than 1, the non‐Newtonian behavior (dilatant) was observed at high shear rates in both formulas of milk. This type of flow behavior is probably related to the formation of an interconnected three‐dimensional network structure by the suspended particles during condensation (Lee & Wagner, 2006). In general, the results of the rheological tests indicated the similarity of both types of milk produced from the two different types of oil. Accordingly, the results showed that the replacement in the oil types did not cause significant effects on the rheological properties of the product.

### 3.7 Milk color

Color is one of the most important commercial attributes of any product which also can be used as an indicator for identifying the defects that occur during the processing. Table 7 shows changes in the color parameters of the two milk powders produced by ostrich and vegetable oil samples. As can be seen, there is no statistical difference between the Lab values of the sample produced from ostrich oil and the vegetable oils (*p* > .05). The lightness value, L* of both powders was close to each other, which indicates that both powders are similar in terms of brightness. O'Shea et al. (2021) analyzed the color values of three types of milk powder and reported that this value ranged between 90 and 95.7, which is close to our findings.

**TABLE 7:** Analysis results of L*, a*, and b* of CIELab color system in powdered milk prepared with ostrich oil and vegetable oils

Milk powder	L*	a*	b*
Ostrich fat	88.5 ± 2.5	−2 ± 1	7 ± 2
Vegetable oil	89.2 ± 3	−3 ± 1	6 ± 3

The high value of redness (a*) in milk powder can indicate a darkening of color and the occurrence of browning reactions due to high temperature in the final stages of drying.

The effect of drying temperature on color values of powdered infant formulas produced by the incorporation of structured lipid was investigated by Nagachinta and Akoh (2013). Their results showed that the redness value increased from −2.28 to −3.12 with increasing the temperature of the spray dryer from 120 to 180, respectively. The variations in the b* value also showed a decreasing trend with increasing drier temperature and were reported as 16.37 and 15.30, respectively.

According to Table 7, the values of parameter b* were positive, which indicates a yellow, cream color. Differences existing between the result of this work and Nagachinta and Akoh (2013) and Zhang et al. (2020) studies on blueness value could be due to the formulation and process parameters.

### 3.8 Sensory evaluation of the product

Sensory evaluation is important in assessing food acceptability as perceived by mothers and infants (Mbela et al., 2018). The results of sensory evaluation by hedonic test are shown in Table 8. Results revealed that the type of fat only had a significant effect on flavor of infant formula and the infant formula produced from ostrich oil accrued fewer scores compared to vegetable oils. Nevertheless, the overall acceptance results showed that there was no difference in acceptance between the two samples; both had a mean acceptance rating of “Like slightly.”

**TABLE 8:** Sensory evaluation results produced infant formula from ostrich oil and vegetable oils

Milk powder	Color	Flavor	Overall acceptance
Ostrich fat	4.6 ± 0.75	3.2 ± 0.49	3.9 ± 0.81
Vegetable oil	4.7 ± 0.54	3.8 ± 0.74	4.2 ± 0.49

## 4 CONCLUSION

In this study, a comparison was made between vegetable oils and ostrich oil and their use as a source of fat in the production of IF. The results showed that major types of fatty acids in all three types of oils were similar to each other and include oleic acid, palmitic acid, and linoleic acid. Despite the similarity of the fatty acid profile between the ostrich oil and breast milk fat, some differences were also revealed especially in lauric and myristic fatty acid contents. While the palmitic acid content which is located in sn‐2 position in triacylglycerol was similar between ostrich oil and vegetable fats, the higher cholesterol level content in ostrich oil increased the similarity between the ostrich oil and breast milk fat. Also, the substitution of ostrich oil instead of vegetable fat sources for infant formulas showed that the chemical composition, rheological properties, color parameters, and sensory properties of the new product did not change considerably and were not statistically significant from each other (*p* > .05). In summary, the results of this study showed that ostrich oil has valuable fatty acids and is very similar to the fatty acid profile of breast milk. However, it is important to note that the amounts of phospholipid (polar lipids) in pure animal fats such as ostrich fat are low, and therefore it can be recommended that blending vegetable oils and ostrich oil may be an effective way to mimic the lipid composition of breast milk. Further research is required to fully investigate this substitution in particular by increasing its palmitic acid content at the sn‐2 position (esterification) and incorporating ARA/DHA in order to deliver a lipid component more similar to that in breast milk.

## FUNDING INFORMATION

This research received no specific grant from any funding agency in the public, commercial, or not‐for‐profit sectors.

## CONFLICT OF INTEREST

The authors declare that there is no conflict of interest.

## ETHICAL APPROVAL

This study does not involve any human or animal testing.

## Data Availability

The data that support the findings of this study are available on request from the corresponding author.
